# Patient Characteristics During Early Transmission of SARS-CoV-2, Palau, January 13–February 24, 2022

**DOI:** 10.3201/eid2909.230182

**Published:** 2023-09

**Authors:** Braiden Eilers, Myra D. Adelbai-Fraser, Johnrey R. Collado, Miriam Van Dyke, Melanie Firestone, Angie S. Guinn, Michael T. Dillon, Richard Brostrom, Michael H. Kinzer, Nick Muñoz, Kazuhiro Okumura, Vance Brown, Oluwatomiloba Ademokun, Ritter Udui, Gaafar J. Uherbelau, W. Thane Hancock

**Affiliations:** Centers for Disease Control and Prevention, Atlanta, Georgia USA (B. Eilers, M. Van Dyke, M. Firestone, A.S. Guinn, M.T. Dillon, R. Brostrom, M.H. Kinzer, V. Brown, O. Ademokun, W.T. Hancock);; Palau Ministry of Health and Human Services, Koror, Palau (M.D. Adelbai-Fraser, J.R. Collado, R. Udui, G.J. Uherbealu);; US Department of Health and Human Services, Washington, DC, USA (N. Muñoz, K. Okumura)

**Keywords:** COVID-19, Palau, hospitalized patients, patient characteristics, SARS-CoV-2, early transmission, Pacific islands, global health, communicable diseases, respiratory infections, zoonoses

## Abstract

Palau had no reported evidence of COVID-19 community spread until January 2022. We chart reviewed hospitalized patients who had a positive SARS-CoV-2 test result during early community transmission. Booster vaccinations and early outpatient treatment decreased hospitalizations. Inadequate hospital infection control practices contributed to iatrogenic COVID-19 and preventable deaths.

Palau is a Pacific Island country that has a population of ≈17,500 persons (*1*). This country has a small health system, remote location, and high prevalence of chronic disease (*2*), which made it exceptionally vulnerable to the effects of COVID-19. Palau took extraordinary steps to prevent the introduction of SARS-CoV-2 by initially closing borders in March 2020 and later transitioning to strict testing and quarantine procedures. The country also expanded testing capacity, maximized vaccinations, and acquired novel COVID-19 therapeutics.

In July 2021, Palau discontinued its mandatory travel quarantine after 95% of the population >18 years of age were fully vaccinated against COVID-19. Limited SARS-CoV-2 infections were soon identified in travelers, but no cases of community transmission were documented until January 13, 2022, when community transmission of SARS-CoV-2 (Omicron BA.1.1) was confirmed. At that time, 98% of the eligible population was fully vaccinated and 31% had received a booster vaccination within the previous 2 months.

Cases increased rapidly (859 in the first 2 weeks), and the first known COVID-19 related hospitalization occurred on January 20, 2022. Rapid antigen testing was offered at a central location and rurally by mobile teams. The Community COVID-19 Care Center (C4) was established to immediately evaluate patients who tested positive for SARS-CoV-2 and, if indicated, provided a novel COVID-19 therapeutic (monoclonal antibody sotrovimab or antiviral drugs molnupiravir or nirmatrelvir/ritonavir) as outpatient treatment. Persons who had abnormal vital signs or severe symptoms were referred to the emergency department.

At Belau National Hospital, the only hospital in Palau, all patients were tested for COVID-19 at admission, and periodic surveillance testing was conducted on patients admitted for non‒COVID-19 health conditions. We examined characteristics of all hospitalized patients who had a positive SARS-CoV-2 test result during the early surge of COVID-19 community transmission, January 13–February 24, 2022. During that period, Palau identified 3,656 patients who had SARS-CoV-2 infection; 57 (1.6%) were hospitalized. We abstracted patient information on demographics, concurrent conditions, vaccination status, oxygen requirement, treatment, and disposition.

Of the 57 hospitalized patients, more were female (32 [56%]) than male (25 [44%]) ; 28 (49%) were >65 years of age. Four (7%) patients were children <5 years of age, including 1 infant born to a mother who had COVID-19 and who tested positive on the first day of life. Fifty-two (91%) patients had >1 known medical condition, putting them at risk for severe COVID-19 (*3*); 29 (51%) patients had >4 risk factors (>65 years of age or medical conditions), putting them at higher risk for severe COVID-19. The 5 (9%) patients who did not have concurrent conditions were the 4 hospitalized children and 1 adult (30‒40 years of age).

Twenty-seven (47%) hospitalized patients were unvaccinated or incompletely vaccinated (5 patients had partial primary vaccination; 4 patients were ineligible for vaccination because they were <5 years old). Twenty (35%) patients had completed their primary vaccination but had not received an appropriate booster (15 patients were eligible for a booster at the time of COVID-19 diagnosis). Ten (18%) had completed their primary vaccination with an appropriate booster (>14 days before COVID-19 diagnosis).

Eighteen (32%) patients required oxygen supplementation during hospitalization. Of those, 4 required high-flow nasal cannula; all were unvaccinated. Although some patients met criteria for intubation, none were mechanically ventilated because of their goals of care.

Seven patients died during hospitalization; 1 death was deemed not related to COVID-19 disease and excluded from the death analysis. Of the 6 (11%) COVID-19 related deaths, 4 (67%) patients were unvaccinated and 2 (33%) had completed primary vaccination but had not received an appropriate booster. All patients who had COVID-19–related deaths had >2 risk factors for developing severe disease. All required oxygen supplementation.

A total of 29 (50%) patients were hospitalized primarily because of COVID-19 pneumonia; 3 of those patients died. Ten patients received remdesivir during their admission. Only 1 patient who received treatment from the C4 returned for admission because of worsening symptoms; that patient survived.

A total of 20 (35%) patients were determined to have hospital-acquired SARS-CoV-2 infection because they tested negative on admission but later tested positive during their hospitalization. Three of those patients died. Eight of the hospital-acquired infections were long-term hospital admissions (Palau has no skilled nursing facilities); 5 patients were unvaccinated, and 1 died.

This analysis characterized hospitalized patients who had SARS-CoV-2 infections in a recently exposed Pacific Islander population that had high rates of chronic illness but excellent COVID-19 immunization coverage and good access to testing and COVID-19 therapeutics. Booster vaccinations appear protective because the risk for hospitalization with COVID-19 was crudely estimated to be 18.6 times higher for unvaccinated persons than for persons who had completed primary vaccination and an appropriate booster. There were no deaths for any of the COVID-19 patients who received novel COVID-19 therapeutics at the C4, suggesting that therapy at time of diagnosis provided additional protection against severe disease. The large proportion of hospital-acquired infections and subsequent preventable deaths highlighted inadequate infection control practices and motivated revision of hospital protocols.
